# A secondary analysis of concurrent use of metformin and tolvaptan in ADPKD tolvaptan trials

**DOI:** 10.1007/s40620-024-01906-x

**Published:** 2024-03-21

**Authors:** I. Kitty Stanley, Anton M. Palma, Andrea K. Viecelli, David W. Johnson, Carmel M. Hawley, Christine E. Staatz, Andrew J. Mallett

**Affiliations:** 1 Centre for Health Services Research, The University of Queensland, Brisbane, QLD Australia; 2 Otsuka Pharmaceutical Development & Commercialization, Inc., Princeton, NJ USA; 3 Department of Kidney and Transplant Services, Princess Alexandra Hospital, Brisbane, Australia; 4 Centre for Kidney Disease Research, The University of Queensland, Brisbane, Australia; 5 School of Pharmacy, The University of Queensland, Brisbane, Australia; 6 Department of Renal Medicine, Townsville University Hospital, Townsville, Australia; 7 College of Medicine and Dentistry, James Cook University, Townsville, Australia; 8 Institute for Molecular Bioscience, The University of Queensland, Brisbane, Australia

Autosomal dominant polycystic kidney disease (ADPKD) is the leading genetic cause of kidney disease [1]. Tolvaptan, a vasopressin V2-receptor antagonist, is the only disease-modifying therapy available with proven efficacy in slowing disease progression. The anti-diabetic medication, metformin, has been theorised to be of potential benefit in ADPKD. Metformin activates 5 AMP-activated protein kinase, which negatively regulates the cystic fibrosis transmembrane conductance regulator and the mammalian target of rapamycin, both of which are implicated in the growth of cysts in ADPKD [2]. Two phase II trials [3, 4] have found metformin to be safe and tolerable in patients with ADPKD, with a non-significant slowing of estimated glomerular filtration rate (eGFR) decline and total kidney volume expansion. A phase III trial is currently underway to further define metformin’s role in ADPKD [5]. Given the established role of tolvaptan and the potential role of metformin in the management of ADPKD, the safety and efficacy of combined therapy are also of interest.

Two phase III trials, Tolvaptan Efficacy and safety in Management of autosomal dominant Polycystic kidney disease and its Outcomes (TEMPO) 3:4 [6] and Replicating Evidence of Preserved Renal function: an Investigation of tolvaptan Safety and Efficacy in ADPKD (REPRISE) [7], established tolvaptan’s efficacy. The datasets of all subjects from these trials were pooled in this analysis and participants were assessed for eligibility for analysis on primary efficacy endpoints. Patients’ use of metformin and allocated treatment groups were used to create 4 groups for analysis: tolvaptan only, placebo only, tolvaptan with metformin and placebo with metformin. Baseline characteristics were described using frequencies (n/%) for categorical variables and descriptive statistics (mean/SE) for continuous variables. Chi-squared tests and t-tests for association assessed whether distributions differed by treatment group. Subject-level incidence of adverse events (n/%) were also compared.

Linear mixed models for repeated measures were conducted to evaluate change in total kidney volume (%) from baseline to 12 and 36 months in the TEMPO 3:4 trial and annualized eGFR slope (mL/min/1.73m^2^ per year) from baseline to 4, 8 and 12 months in both trials. Due to varying eligibility criteria in each trial, analyses for eGFR slope were restricted to participants with stage 2 chronic kidney disease (CKD) or higher at baseline. Both models included fixed effects for visit (categorical), treatment group and a visit*treatment interaction term and random effects for repeated measures within subjects. Model-estimated least square means and 95% confidence intervals for each endpoint were calculated at each visit and compared between each pair of treatment groups, adjusted for multiple comparisons using Tukey’s Honest Significant Difference test. Analyses were repeated with baseline CKD stage. R statistical software v4.2.1 (R Core Team, Vienna, Austria, 2020) was used for the analyses.

The pooled dataset included 2488 subjects from the TEMPO 3:4 (*n* = 1157) and REPRISE (*n* = 1331) trials. Metformin use was observed in 28 participants and used to create 4 treatment groups for analysis: tolvaptan only (*n* = 1390), placebo only (*n* = 1070), tolvaptan with metformin (*n* = 18), placebo with metformin (*n* = 10). Baseline characteristics by treatment group are shown in Supplementary Table S1. At baseline, metformin users were older (47 vs. 43.5 years), heavier (96 vs. 81 kg), had lower eGFR (49.6 vs. 58 mL/min/1.73m^2^) and higher prevalence of cardiovascular-related comorbidities, including diabetes mellitus (79 vs. 1.5%) (*p* < 0.001).

Results from models of the change in eGFR are displayed in Fig. 1a. Baseline kidney function was measured at the end of the titration phase and excluded any observations after end of treatment to minimize the potential acute haemodynamic effects of tolvaptan. Participants receiving tolvaptan had a slightly slower decline in kidney function vs. placebo only (annualized eGFR slope – 3.2% [95%CI, – 4.1 to – 2.4%] vs. – 4.0%, [95%CI, – 5 to – 3.1%] *p* = 0.584). Participants receiving both tolvaptan and metformin did not experience a statistically significant difference in eGFR decline compared with the other groups (Supplemental Table S2). Results also did not differ by CKD stage at baseline (Fig. 1b, Supplemental Table S3).

**Fig. 1:**
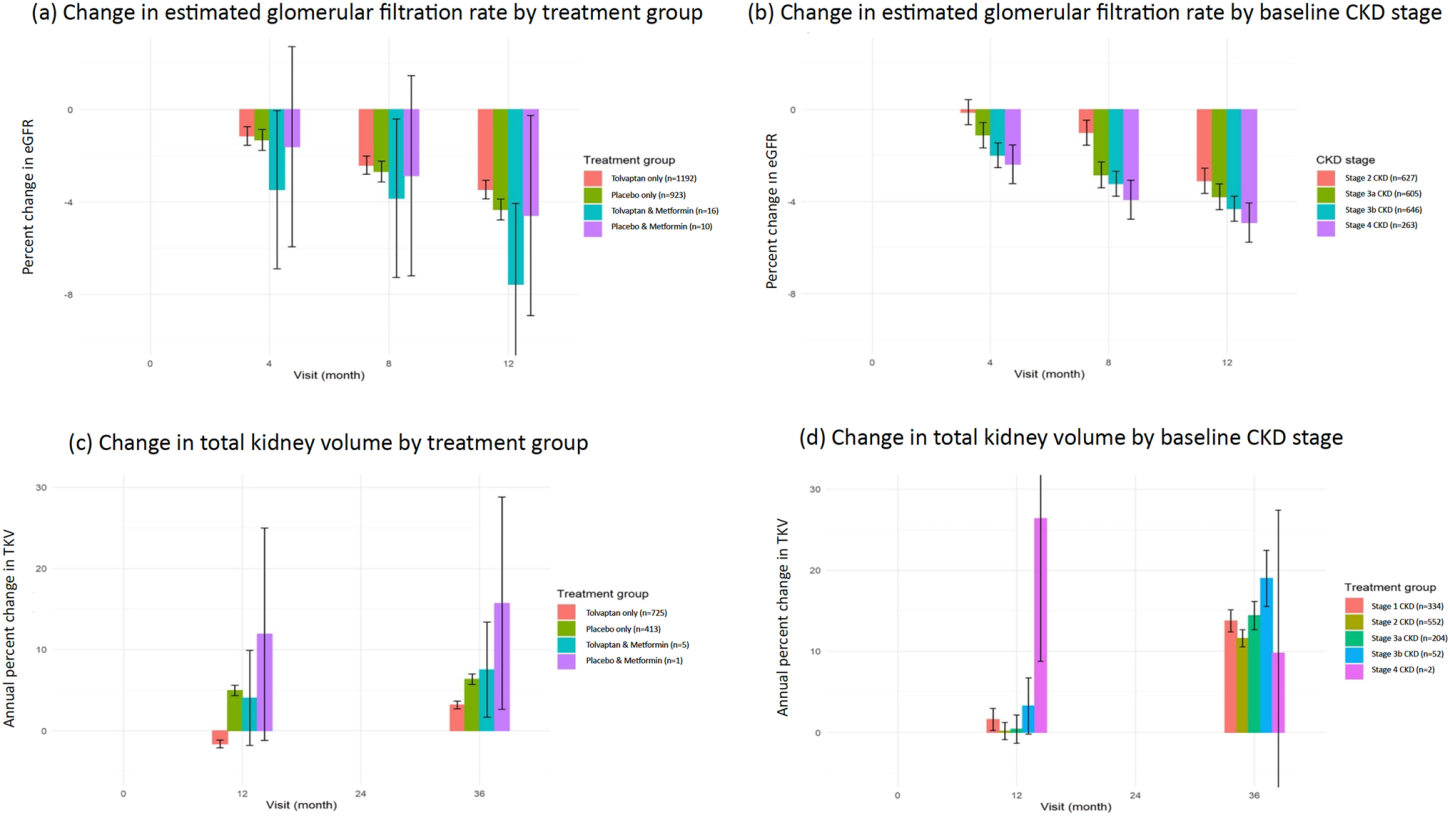
Change in estimated glomerular filtration rate and total kidney volume by treatment group and CKD stage

Total kidney volume increased significantly from baseline to 36 months in all treatment groups in the TEMPO 3:4 trial, tolvaptan alone (3.2%, 95%CI, 2.7–3.7%), placebo (6.3%, 95%CI, 5.7–7.0%), metformin with tolvaptan (7.5%, 95%CI, 1.7% to 13.4%) and metformin with placebo (15.7%, 95%CI, 2.6–28.8%). The only statistically significant difference was between tolvaptan only and placebo only, *p* =  < 0.001; (Fig. 1c). Results stratified by CKD stage showed no clear differences (Fig. 1d, Supplemental Table S3).

The most common adverse events experienced during the trials were polyuria (25.8% of overall participants), thirst (20.1%) and headache (14.5%). Adverse events and safety outcomes were comparable between metformin users and non-users (Supplemental Table S4). There were higher rates of urinary tract infection (20%) and lower rates of nausea (0%) in the metformin with placebo group (p-value 0.04), although this was the smallest participant group.

This preliminary report analysed outcomes from the TEMPO3:4 and REPRISE trials in participants using metformin at baseline. Efficacy estimates showed no meaningful difference between participants treated with metformin. Concomitant therapy showed no signal of harm and further investigation of metformin combined with tolvaptan is warranted. The upcoming phase III trial of metformin for ADPKD will permit the inclusion of patients being treated with tolvaptan [5], and an active comparator trial comparing metformin to tolvaptan in ADPKD has been proposed [8].

Limitations of this study include the small sample size of metformin-treated participants, which restricts statistical power. Neither TEMPO3:4 nor REPRISE excluded participants with type 2 diabetes mellitus. Unsurprisingly, diabetes was identified as a comorbidity in 80% of metformin-treated participants, compared to just 1.5% of participants not on metformin, and may have confounded these results (Supplementary Table S1). To create a unified data set in the models for eGFR slope, participants from the REPRISE population with stage 1 CKD were excluded, and TEMPO 3:4 results were restricted to 12 months. This may explain why we observed non-significant differences in eGFR decline between tolvaptan- and placebo-treated participants, whereas statistically significant benefits were reported in the primary publications of these trials [6, 7].

Overall, these data suggest that metformin may be safe to use in conjunction with tolvaptan. Due to the low number of participants taking metformin, there are currently insufficient data to determine the impact of co-therapy on clinical outcomes. Upcoming trials may provide more insight.

## Supplementary Information

Below is the link to the electronic supplementary material.

Supplementary file1 (DOCX 40 kb)

## Data Availability

Data may be made available upon request and application to Otsuka Pharmaceutical Development & Commercialization, Inc.
