# Supplementary material for: A secondary analysis of concurrent use of metformin and tolvaptan in ADPKD tolvaptan trials
Source: J Nephrol. 2024 Mar 21;37(5):1417–9. doi: 10.1007/s40620-024-01906-x (PMC11405464; doi:10.1007/s40620-024-01906-x)
Supplement: Supplementary file 1 — Supplementary file1 (DOCX 40 kb) [file 40620_2024_1906_MOESM1_ESM.docx]

**Supplementary**

**Supplementary Table S1**: Baseline characteristics by treatment group of included participants. values presented as n (%) or mean (SE).

|  |  | **TREATMENT GROUP** | | | |  |
| --- | --- | --- | --- | --- | --- | --- |
| **Characteristic** | **Category** | **Tolvaptan only (n=1,390)** | **Placebo only (n=1,070)** | **Tolvaptan + Metformin (n=18)** | **Metformin only (n=10)** | **p-value^1^** |
|  |  |  |  |  |  |  |
| Sex | Female | 667 (48.0%) | 541 (50.6%) | 10 (55.6%) | 2 (20.0%) | 0.152 |
|  | Male | 723 (52.0%) | 529 (49.4%) | 8 (44.4%) | 8 (80.0%) |  |
| Age (years) |  | 42.9 (8.6) | 44.1 (8.6) | 44.9 (7.4) | 49.4 (7.9) | <0.001 |
| Race | White | 1225 (88.1%) | 948 (88.6%) | 14 (77.8%) | 9 (90.0%) | 0.745 |
|  | Asian | 112 (8.1%) | 75 (7.0%) | 3 (16.7%) | 1 (10.0%) |  |
|  | Black or African American | 33 (2.4%) | 25 (2.3%) | 1 (5.6%) | 0 (0.0%) |  |
|  | Other | 20 (1.4%) | 22 (2.1%) | 0 (0.0%) | 0 (0.0%) |  |
| Region | Non-US | 868 (62.4%) | 663 (62.0%) | 10 (55.6%) | 4 (40.0%) | 0.479 |
|  | US | 522 (37.6%) | 407 (38.0%) | 8 (44.4%) | 6 (60.0%) |  |
| Height (cm) |  | 173.7 (10.3) | 173.0 (10.0) | 170.4 (10.6) | 175.5 (10.1) | <0.001 |
| Weight (kg) |  | 81.6 (18.8) | 80.7 (18.5) | 89.4 (22.5) | 102.8 (34.9) | <0.001 |
| eGFR at baseline (mL/min/1.73m**^2^**) |  | 59.2 (24.5) | 56.8 (25.4) | 56.1 (16.8) | 43.1 (9.8) | <0.001 |
| CKD stage | Stage 1 | 189 (13.7%) | 144 (13.5%) | 1 (5.9%) | 0 (0.0%) | 0.003 |
|  | Stage 2 | 386 (28.0%) | 237 (22.2%) | 4 (23.5%) | 0 (0.0%) |  |
|  | Stage 3a | 342 (24.8%) | 253 (23.7%) | 7 (41.2%) | 3 (30.0%) |  |
|  | Stage 3b | 322 (23.3%) | 313 (29.3%) | 5 (29.4%) | 6 (60.0%) |  |
|  | Stage 4 | 142 (10.3%) | 120 (11.2%) | 0 (0.0%) | 1 (10.0%) |  |
| Comorbidities | Dyslipidemia | 198 (14.2%) | 170 (15.9%) | 6 (33.3%) | 7 (70.0%) | <0.001 |
|  | Cholesterolemia | 117 (8.4%) | 78 (7.3%) | 2 (11.1%) | 1 (10.0%) | 0.716 |
|  | Diabetes | 22 (1.6%) | 15 (1.4%) | 14 (77.8%) | 8 (80.0%) | <0.001 |
|  | Obesity | 50 (3.6%) | 48 (4.5%) | 1 (5.6%) | 2 (20.0%) | 0.048 |
|  | Depression | 103 (7.4%) | 88 (8.2%) | 1 (5.6%) | 1 (10.0%) | 0.861 |
|  | Hypertension | 690 (49.6%) | 668 (62.4%) | 13 (72.2%) | 9 (90.0%) | <0.001 |
| Study participant number | 156-04-251 (TEMPO 3:4) | 734 (52.8%) | 416 (38.9%) | 6 (33.3%) | 1 (10.0%) | <0.001 |
|  | 156-13-210  (REPRISE) | 656 (47.2%) | 654 (61.1%) | 12 (66.7%) | 9 (90.0%) |  |
| ^1^ Sample characteristics between treatment groups were compared using chi-squared tests for categorical variables and t-tests for continuous variables | | | | | | |
| CKD, chronic kidney disease; eGFR, estimated glomerular filtration rate; n, number; SE, standard error; US, United States. | | | | | | |

**Supplementary Table S2:** Change in total kidney volume and estimated glomerular filtration rate of participants by treatment group.

|  | **TOTAL KIDNEY VOLUME (TEMPO 3:4 ONLY)** | **p-value^1^** | | | |
| --- | --- | --- | --- | --- | --- |
| **By treatment group** | **% change/ year (95%CI)** | **vs. Baseline** | **vs. Tolvaptan only** | **vs. Placebo only** | **vs. Metformin only** |
| **12 months** |  |  |  |  |  |
| Tolvaptan only (n=725) | -1.6%  (-2.1%, -1.1%) | <0.001 |  |  |  |
| Placebo only (n=413) | 5.0%  (4.3%, 5.6%) | <0.001 | <0.001 |  |  |
| Tolvaptan + metformin (n=5) | 4.0%  (-1.8%, 9.9%) | 0.366 | 0.079 | 0.611 |  |
| Placebo + metformin (n=1) | 11.9%  (-1.2%, 25.0%) | 0.176 | 0.113 | 0.984 | 0.583 |
| **36 months** |  |  |  |  |  |
| Tolvaptan only (n=725) | 3.2%  (2.7%, 3.7%) | <0.001 |  |  |  |
| Placebo only (n=413) | 6.3%  (5.7%, 7.0%) | <0.001 | <0.001 |  |  |
| Tolvaptan + metformin (n=5) | 7.5%  (1.7%, 13.4%) | 0.032 | 0.119 | 0.349 |  |
| Placebo + metformin (n=1) | 15.7%  (2.6%, 28.8%) | 0.049 | 0.316 | 0.967 | 0.549 |
|  | **eGFR slope (TEMPO 3:4 & REPRISE)** | **p-value^1^** | | | |
| **By treatment group** | **% change / year**  **(95% CI)** | **vs. Baseline** | **vs. Tolvaptan only** | **vs. Placebo only** | **vs. Metformin only** |
| **12 months** |  |  |  |  |  |
| Tolvaptan only (n=1,192) | -3.2%  (-4.1%, -2.4%) | <0.001 |  |  |  |
| Placebo only (n=923) | -4.0%  (-5%, -3.1%) | <0.001 | 0.584 |  |  |
| Tolvaptan + metformin (n=16) | -7.0%  (-14.7%, 0.7%) | 0.279 | 0.938 | 0.883 |  |
| Placebo + metformin (n=10) | -3.8%  (-13.3%, 5.7%) | 0.861 | 0.739 | 0.854 | 0.708 |
| ^1^ p-values represent comparisons of least square means within group (1st column) or between groups (columns 2-3) at a given visit vs. baseline mean, adjusted for multiple comparisons using Tukey's Honest Significant Difference test.  CI, confidence interval; n, number. | | | | | |

**Supplementary Table S3:** Change in total kidney volume and estimated glomerular filtration rate of participants by stage of chronic kidney disease

|  | **TOTAL KIDNEY VOLUME (TEMPO 3:4 ONLY)** | **p-value^1^** | | | | | | | |
| --- | --- | --- | --- | --- | --- | --- | --- | --- | --- |
| **By CKD stage at baseline** | **% change / year**  **95% CI** | **vs. Baseline** | **vs. Stage 1** | **vs.**  **Stage 2** | | **vs. Stage 3a** | | **vs. Stage 3b** | |
| **12 months** |  |  |  |  | |  | |  | |
| Stage 1 | 1.6%  (0.2%, 3.0%) | 0.057 |  |  | |  | |  | |
| Stage 2 | 0.2%  (-0.9%, 1.2%) | 0.947 | 0.306 |  | |  | |  | |
| Stage 3a | 0.4%  (-1.3%, 2.2%) | 0.886 | 0.728 | 0.999 | |  | |  | |
|  |  |  |  |  | |  | |  | |
| Stage 3b | 3.3%  (-0.2%, 6.7%) | 0.153 | 0.834 | 0.269 | | 0.426 | |  | |
| Stage 4 | 26.4%  (8.8%, 44.0%) | 0.009 | 0.010 | 0.005 | | 0.006 | | 0.023 | |
| **36 months** |  |  |  |  | |  | |  | |
| Stage 1 | 13.8%  (12.4%, 15.1%) | <0.001 |  |  | |  | |  | |
| Stage 2 | 11.6%  (10.5%, 12.7%) | <0.001 | 0.031 |  | |  | |  | |
| Stage 3a | 14.4%  (12.7%, 16.2%) | <0.001 | 0.962 | 0.013 | |  | |  | |
| Stage 3b | 19.0%  (15.5%, 22.5%) | <0.001 | 0.010 | <0.001 | | 0.049 | |  | |
| Stage 4 | 9.8%  (-7.8%, 27.4%) | 0.519 | 0.985 | 0.999 | | 0.974 | | 0.757 | |
|  | **eGFR slope (TEMPO 3:4 & REPRISE)** | **p-value^1^** | | | | | | | |
| **By CKD stage at baseline** | **% change / year**  **95% CI** | **vs. Baseline** | **-** | | **vs.**  **Stage 2** | | **vs. Stage 3a** | | **vs. Stage 3b** |
| **12 months** |  |  |  |  | |  | |  | |
| Stage 2 | -3.2%  (-4.4%, -2.0%) | <0.001 | - |  | |  | |  | |
| Stage 3a | -3.5%  (-4.7%, -2.3%) | <0.001 | - | 0.979 | |  | |  | |
| Stage 3b | -3.8%  (-5.0%, -2.6%) | <0.001 | - | 0.828 | | 0.969 | |  | |
| Stage 4 | -4.4%  (-6.2%, -2.5%) | <0.001 | - | 0.606 | | 0.791 | | 0.936 | |
| ^1^ p-values represent comparisons of least square means within group (1st column) or between groups (columns 2-3) at a given visit vs. baseline mean, adjusted for multiple comparisons using Tukey's Honest Significant Difference test.  CI, confidence interval; CKD, chronic kidney disease | | | | | | | | | |

**Supplementary Table S4:** Frequencies of adverse events of therapy by treatment group.

|  | Tolvaptan | | Placebo | |  |
| --- | --- | --- | --- | --- | --- |
| **Adverse event** | **No metformin**  (n=1390) | **Metformin**  (n=13) | **No metformin**  (n=1070) | **Metformin**  (n=10) | **p- value** |
| Polyuria | 414 (29.8%) | 5 (27.8%) | 219 (20.5%) | 3 (30%) | 0.15 |
| Thirst | 328 (23.6%) | 3 (16.7%) | 167 (15.6%) | 2 (20%) | 0.12 |
| Headache | 218 (15.7%) | 1 (5.6%) | 141 (13.2%) | 1 (10%) | 0.77 |
| Nocturia | 200 (14.4%) | 0 (0%) | 121 (11.3%) | 1 (10%) | 0.45 |
| Dry mouth | 168 (12.1%) | 1 (5.6%) | 106 (9.9%) | 1 (10%) | 0.92 |
| Fatigue | 130 (9.4%) | 0 (0%) | 61 (5.7%) | 0 (0%) | 0.11 |
| Worsening hypertension | 128 (9.2%) | 1 (5.6%) | 90 (8.4%) | 3 (30%) | 0.09 |
| Polydipsia | 121 (8.7%) | 1 (5.6%) | 74 (6.9%) | 0 (0%) | 0.82 |
| Nausea | 105 (7.6%) | 1 (5.6%) | 98 (9.2%) | 0 (0%) | 0.04 |
| Urinary tract infection | 99 (7.1%) | 2 (11.1%) | 89 (8.3%) | 2 (20%) | 0.04 |
| Worsening kidney pain | 98 (7.1%) | 3 (16.7%) | 71 (6.6%) | 0 (0%) | 0.28 |
| n=number. p-value corresponds to global chi-square test of association across all four treatment groups. | | | | | |
